# Synthesis and biological evaluation of novel 8-substituted quinoline-2-carboxamides as carbonic anhydrase inhibitors

**DOI:** 10.1080/14756366.2019.1626376

**Published:** 2019-06-20

**Authors:** Pavitra S. Thacker, Pirpasha Shaikh, Andrea Angeli, Mohammed Arifuddin, Claudiu T. Supuran

**Affiliations:** a Department of Medicinal Chemistry, National Institute of Pharmaceutical Education and Research (NIPER), Hyderabad, India;; b Neurofarba Department, Section of Pharmaceutical Chemistry, University of Florence, Florence, Italy

**Keywords:** Carbonic anhydrases, metalloenzyme, sulfonamides, quinoline, tail approach

## Abstract

A series of novel 8-substituted-N-(4-sulfamoylphenyl)quinoline-2-carboxamides was synthesised by the reaction of 8-hydroxy-N-(4-sulfamoylphenyl) quinoline-2-carboxamide with alkyl and benzyl halides. The compounds were assayed for carbonic anhydrase (CA) inhibitory activity against four hCA isoforms, hCA I, hCA II, hCA IV, and hCA IX. Barring hCA IX, all the isoforms were inhibited from low to high nanomolar range. hCA I was inhibited in the range of 61.9–8126 nM, with compound **5h** having an inhibition constant of *K*_I_ = 61.9 nM. hCA II was inhibited in the range of 33.0–8759 nM, with compound **5h** having an inhibition constant of 33.0 nM and compounds **5a** and **5b** having inhibition constants of 88.4 and 85.7 nM, respectively. hCA IV was inhibited in the range of 657.2–6757 nM. Hence, compound **5h**, possessing low nanomolar hCA I and II inhibition, can be selected as a lead for the design of novel CA I and II inhibitors.

## Graphical Abstract

**Figure:**
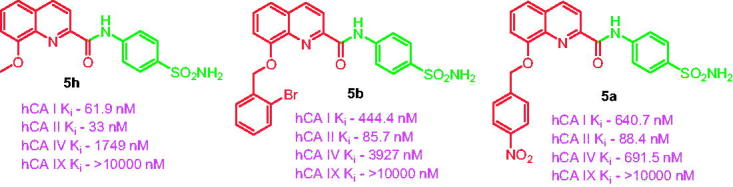

## 1. Introduction

Carbonic anhydrases (CAs, EC 4.2.1.1) are a group of convergently evolved, ubiquitous metalloenzymes, which play a pivotal role in the maintenance of pH homoeostasis across all living phyla^1–4^. They catalyse the reversible interconversion of carbon dioxide to bicarbonate and protons under physiological conditions via a ping pong mechanism, which is normally very slow under non-catalytic conditions (reaction *1*): 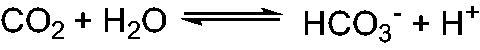
   (1)

By efficiently catalysing the above reaction, CAs play an important role in many metabolic processes like facilitating the transport of CO_2_ between the metabolising tissues and lungs, lipogenesis, gluconeogenesis, and ureagenesis^1–4^.

Up until now, eight genetically distinct families of CAs are known to be present across all phyla. These seven families are α-, β-, γ-, δ-, ζ-, η-, θ-, and the recently reported ι-class^5^. The metal ion at the active site, which plays a highly important role in the catalytic activity of the enzyme, is variable among the different families, and can be Zn(II), Cd(II), Fe(II), and even Mn(II) in the ι-CAs^1–5^. The α-CAs are predominantly found in vertebrates, protozoa, algae, bacteria, and the cytoplasm of green plants. The β-CAs are bacteria, algae, fungi, some archaea, and the chloroplasts of monocots and dicots. The γ-CAs are found in archaea and bacteria. The δ-, ζ-, -, θ-, and ι-CAs are present in marine diatoms. The η-CAs are present in various *Plasmodium* species^5^.

α-CAs are widely distributed in many organisms and their number of isoforms is rather high, with 15 such CAs being present in humans and other primates and 16 in other mammals. In humans, the sub-cellular localisation of the isoforms is as follows: CA I, CA II, CA III, CA VII, CA VIII, CA X, CA XI, and CA XIII are cytosolic, CA IV, CA IX, CA XII, CAX IV, and CA XV are membrane-bound, CA VA and VB are mitochondrial, whereas CA VI is secreted in milk and saliva^1–4^. Different isoforms are implicated in different diseases; hence, selective inhibition of a particular isoform may lead to the rectification of that particular disease in which it plays a major role^1–4^.

**Figure 1.**
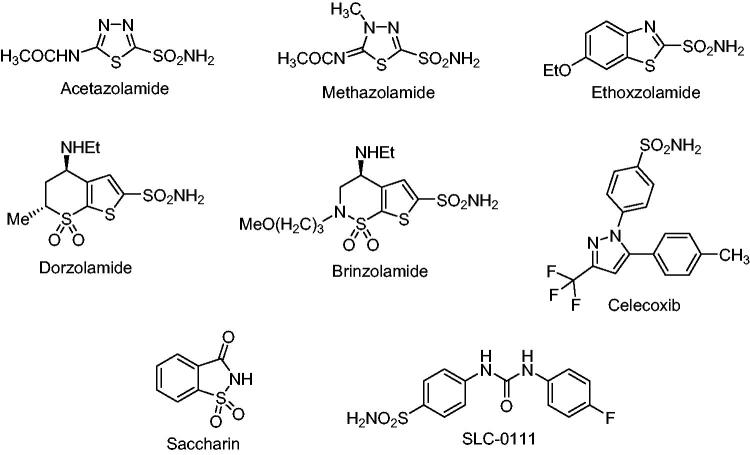
Structures of some clinically used sulfonamides.

Sulfonamides and their isosteres (sulfamates and sulfamides) have been known for many years for their effective inhibition of many CA isoforms^5–8^. Their mode of inhibition is by binding to the metal ion present in the active site, in a deprotonated form, as sulfonamidate anion. About 20 compounds incorporating the sulfonamide moiety are in clinical use for many years, with one of the compounds, SLC-0111, developed by one of our groups, being in phase II clinical trials. Some of the sulfonamide molecules in clinical use since many years are shown in Figure 1^6–11^.

**Figure 2.**
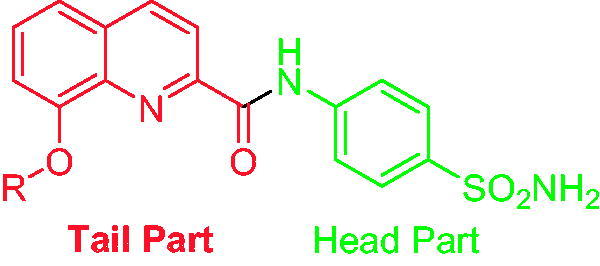
The “Tail approach” method for the design of novel CA inhibitors in this work.

Quinoline is an aromatic, heterocyclic, nitrogen containing compound which is having a benzene ring fused with a pyridine ring at two adjacent carbon atoms^12^. The quinoline nucleus exhibit diverse biological activities such as anticancer, antimalarial, antitubercular, antibacterial, antiprotozoal, antiproliferative, anti-inflammatory, antihypertensive, and anti-HIV activity^13^. One of the potent quinoline derivatives is 8-hydroxy quinoline. It is obtained from plants as well as by synthesis. It is basically a small, planar and lipophilic molecule having an array of biological activities and also good metal chelating properties^14^. Supuran and coworkers have previously investigated the quinoline scaffold, wherein they found it to exhibit potent activities on various CA isoforms^15–17^. Hence, in order to further probe the efficacy of the quinoline scaffold for CA inhibition, the “tail approach” (Figure 2) was adopted and novel 8-substituted-N-(4-sulfamoylphenyl) quinoline-2-carboxamides were synthesised and assayed for their CA inhibitory activity against four CA isoforms, namely, CA I, II, IV, and IX. The drug acetazolamide (AAZ) was used as a drug standard.

## 2. Materials and methods; chemistry part

### 2.1. General

All the chemicals and solvents were procured and utilised as such from the suppliers. Wherever necessary, anhydrous solvents were used. Thin layer chromatography (TLC) analysis was done by utilising Merck silica gel 60 F_254_ aluminium plates. Stuart digital melting point apparatus (SMP 30) was used in determining the melting points of the compounds, which are uncorrected. ^1^H and ^13^C NMR spectra were recorded using Bruker Avance 500 MHz and 125 MHz respectively using DMSO-d_6_ as the solvent. Chemical shift values are recorded in ppm using TMS as the internal standard. HRMS were determined by Agilent QTOF mass spectrometer 6540 series instrument and were performed using ESI techniques at 70 eV.

#### 2.1.1. General procedure for the preparation of 8-hydroxy-N-(4-sulfamoylphenyl)quinoline-2-carboxamide

To a stirred solution of intermediate **3** (1 g, 5 mmol 1 eq.) in dry DMF (10 ml) HATU was added (3 g, 7.9 mmol, 1.5 eq.) at 0 °C. The resultant solution was stirred for one hour at 0 °C. Thereafter, sulfanilamide (1 g, 5.5 mmol, 1.1 eq.) and DIPEA (2 g, 15 mmol, 3 eq.) were added to the reaction mixture and the resultant solution was allowed to stir for overnight at room temperature. The completion of the reaction was monitored by TLC. On completion of the reaction mixture as evidenced by the TLC, it was dumped into crushed ice. The precipitated solid was collected by filtration and it was subjected to column chromatography using silica gel 60–120 mesh as the stationary phase and EtOAc:hexane 6:4 as mobile phase to afford intermediate **4** as a beige solid. Yield (60%).

#### 2.1.2. General procedure for the preparation of 8-substituted-N-(4-sulfamoylphenyl)quinoline-2-carboxamidederivatives (5a–h)

To a stirred solution of intermediate **4** (80 mg, 0.2 mmol, 1 eq.) in acetone (5 ml) K_2_CO_3_ was added (22 mg, 0.4 mmol, 2 eq.) and the resultant solution was allowed to stir for 15 min. Thereafter, alkyl or benzyl halide (1.5 eq.) was added to the reaction mixture and it was allowed to stir for overnight at room temperature. The completion of the reaction was monitored by TLC. The reaction solvent was distilled off under vacuum and the crude residue was subjected to column chromatography using silica gel 60–120 mesh as the stationary phase and EtOAc:hexane 4:6 as mobile phase to afford the final compounds **5a**–**h**.

#### 2.1.3. 8-((4-Nitrobenzyl)oxy)-N-(4-sulfamoylphenyl)quinoline-2-carboxamide (5a)

Yellow solid, yield: 55%; mp: 257–259 °C; ^1^H NMR (500 MHz, DMSO) *δ* 10.78 (s, 1H), 8.62 (d, *J* = 8.5 Hz, 1H), 8.31 (d, *J* = 8.5 Hz, 2H), 8.26 (d, *J* = 8.5 Hz, 1H), 8.01 (d, *J* = 8.6 Hz, 2H), 7.97 (d, *J* = 8.4 Hz, 2H), 7.90 (d, *J* = 8.6 Hz, 2H), 7.68 (d, *J* = 7.0 Hz, 2H), 7.42 (d, *J* = 6.0 Hz, 1H), 7.35 (s, 2H), 5.62 (s, 2H). ^13^C NMR (125 MHz, DMSO) *δ* 163.46, 154.24, 148.83, 147.53, 145.62, 141.35, 139.69, 138.78, 138.45, 130.82, 129.49, 128.39, 127.34, 124.04, 120.81, 119.86, 119.69, 112.24, 69.58. HRMS (ESI): *m/z* calculated for C_23_H_18_N_4_O_6_S 479.1025, found 479.1030 [M + H]^+^.

#### 2.1.4. 8-((2-Bromobenzyl)oxy)-N-(4-sulfamoylphenyl)quinoline-2-carboxamide (5b)

Yellow solid, yield: 40%; mp: 270–272 °C ^1^H NMR (500 MHz, DMSO) *δ* 10.76 (s, 1H), 8.62 (d, *J* = 8.5 Hz, 1H), 8.25 (d, *J* = 8.5 Hz, 1H), 7.98 (d, *J* = 8.7 Hz, 2H), 7.92–7.85 (m, 2H), 7.68 (d, *J* = 6.4 Hz, 2H), 7.65 (s, 3H), 7.41 (dd, *J* = 6.2, 2.6 Hz, 1H), 7.33 (s, 2H), 5.45 (s, 2H). ^13^C NMR (125 MHz, DMSO) *δ* 162.43, 153.40, 147.68, 140.27, 138.61, 137.71, 137.46, 136.14, 130.76, 129.73, 128.78, 128.47, 126.30, 120.25, 119.52, 118.71, 118.53, 111.26, 68.88. HRMS (ESI): *m/z* calculated for C_23_H_18_BrN_3_O_4_S 512.0280, found 514.0265 [M + 2]^+^.

#### 2.1.5. 8-(Benzyloxy)-N-(4-sulfamoylphenyl)quinoline-2-carboxamide (5c)

Yellow solid, yield: 60%; mp: 247–249 °C^1^H NMR (500 MHz, DMSO) *δ* 10.79 (s, 1H), 8.60 (t, *J* = 8.4 Hz, 1H), 8.24 (d, *J* = 8.4 Hz, 1H), 7.97 (d, *J* = 8.6 Hz, 2H), 7.91 (d, *J* = 8.6 Hz, 2H), 7.70 (d, *J* = 7.4 Hz, 2H), 7.67 (d, *J* = 4.2 Hz, 2H), 7.47 (t, *J* = 7.4 Hz, 2H), 7.44–7.41 (m, 1H), 7.40 (d, *J* = 7.4 Hz, 1H), 7.35 (s, 2H), 5.46 (s, 2H). ^13^C NMR (125 MHz, DMSO) *δ* 163.40, 154.65, 148.55, 141.33, 139.67, 138.75, 138.53, 137.72, 129.58, 128.91, 128.27, 127.64, 127.41, 120.70, 120.39, 119.61, 119.48, 112.14, 70.64, 48.96. HRMS (ESI): *m/z* calculated for C_23_H_19_N_3_O_4_S434.1175, found 434.1175 [M + H]^+^.

#### 2.1.6. 8-((3,5-Dimethylbenzyl)oxy)-N-(4-sulfamoylphenyl)quinoline-2-carboxamide (5d)

Yellow solid, yield: 50%; mp: 290–292 °C ^1^H NMR (500 MHz, DMSO) *δ* 10.79 (s, 1H), 8.61 (d, *J* = 8.5 Hz, 1H), 8.24 (d, *J* = 8.4 Hz, 1H), 7.97 (d, *J* = 8.6 Hz, 2H), 7.87 (d, *J* = 8.7 Hz, 2H), 7.69–7.66 (m, 2H), 7.42 (dd, *J* = 5.7, 2.7 Hz, 1H), 7.35 (s, 2H), 7.30 (s, 2H), 7.01 (s, 1H), 5.35 (s, 2H), 2.32 (s, 6H), 2.09 (s, 3H).^13^C NMR (125 MHz, DMSO) *δ* 163.45, 154.72, 148.58, 141.36, 139.66, 138.77, 138.47, 137.96, 137.48, 130.79, 129.65, 127.27, 125.24, 120.36, 119.67, 119.54, 112.01, 70.75, 21.43. HRMS (ESI): *m/z* calculated for C_25_H_23_N_3_O_4_S462.1488, found 462.1492 [M + H]^+^.

#### 2.1.7. 8-((3-Chlorobenzyl)oxy)-N-(4-sulfamoylphenyl)quinoline-2-carboxamide (5e)

Yellow solid, yield: 60%; mp: 279–281 °C ^1^H NMR (500 MHz, DMSO) *δ* 10.85 (s, 1H), 8.66 (d, *J* = 8.2 Hz, 1H), 8.29 (d, *J* = 8.2 Hz, 1H), 8.06 (d, *J* = 8.0 Hz, 2H), 7.93 (d, *J* = 8.9 Hz, 3H), 7.73 (s, 2H), 7.66 (d, *J* = 6.7 Hz, 1H), 7.58–7.45 (m, 3H), 7.39 (s, 2H), 5.50 (s, 2H). ^13^C NMR (125 MHz, DMSO) *δ* 163.36, 154.41, 148.62, 141.31, 140.30, 139.66, 138.77, 138.42, 133.76, 130.79, 129.56, 128.18, 127.31, 127.21, 126.09, 120.59, 119.68, 119.55, 112.01, 69.69.

#### 2.1.8. 8-((2,5-Difluorobenzyl)oxy)-N-(4-sulfamoylphenyl)quinoline-2-carboxamide (5f)

Yellow solid, yield: %;65 mp: 260–262 °C ^1^H NMR (500 MHz, DMSO) *δ* 10.84 (s, 1H), 8.63 (d, *J* = 8.5 Hz, 1H), 8.25 (d, *J* = 8.5 Hz, 1H), 8.02 (d, *J* = 8.7 Hz, 2H), 7.87 (d, *J* = 8.6 Hz, 4H), 7.71 (d, *J* = 6.7 Hz, 2H), 7.51 (dd, *J* = 6.5, 2.2 Hz, 1H), 7.35–7.31 (m, 3H), 5.49 (s, 2H). ^13^C NMR (125 MHz, DMSO) *δ* 163.38, 159.82, 157.91, 157.19, 155.27, 154.19, 148.75, 141.38, 139.66, 138.81, 138.49, 130.77, 129.57, 127.29, 120.98, 119.59, 117.45, 117.19, 116.62, 116.16, 112.48, 64.56. HRMS (ESI): *m/z* calculated for C_23_H_17_F_2_N_3_O_4_S 470.0986, found 470.0994 [M + H]^+^.

#### 2.1.9. 8-(Prop-2-yn-1-yloxy)-N-(4-sulfamoylphenyl)quinoline-2-carboxamide (5g)

Yellow solid, yield: 30%; mp: 240–242 °C ^1^H NMR (500 MHz, DMSO) *δ* 10.76 (s, 1H), 8.62 (d, *J* = 8.5 Hz, 1H), 8.24 (d, *J* = 8.5 Hz, 1H), 8.07 (d, *J* = 8.7 Hz, 2H), 7.89 (t, *J* = 8.7 Hz, 2H), 7.74–7.70 (m, 2H), 7.49–7.43 (m, 1H), 7.33 (s, 2H), 5.17 (d, *J* = 2.1 Hz, 2H), 3.67 (t, *J* = 2.1 Hz, 1H). ^13^C NMR (125 MHz, DMSO) *δ* 163.79, 153.50, 149.21, 141.47, 139.69, 138.72, 138.51, 130.78, 129.25, 127.22, 121.08, 120.26, 119.81, 112.64, 79.58, 79.34, 57.15. HRMS (ESI): *m/z* calculated for C_19_H_15_N_3_O_4_S 382.0862, found 404.0684 [M + Na]^+^.

#### 2.1.10. 8-Methoxy-N-(4-sulfamoylphenyl)quinoline-2-carboxamide (5h)

Yellow solid, yield: 50%; mp: 261–263 °C; ^1^H NMR (500 MHz, DMSO) *δ* 10.77 (s, 1H), 8.59 (d, *J* = 8.5 Hz, 1H), 8.23 (d, *J* = 8.4 Hz, 1H), 8.10 (dd, *J* = 18.5, 8.6 Hz, 2H), 7.85 (dd, *J* = 24.8, 8.6 Hz, 2H), 7.72–7.63 (m, 2H), 7.40–7.31 (m, 3H), 4.08 (s, 3H). ^13^C NMR (125 MHz, DMSO) *δ* 163.82, 155.77, 148.91, 141.50, 139.65, 138.55, 138.25, 130.67, 129.59, 128.29, 127.20, 120.49, 120.27, 119.92, 119.76, 109.97, 56.44. HRMS (ESI): *m/z* calculated for C_17_H_15_N_3_O_4_S 358.0862, found 358.0865 [M + H]^+^. Spectral data are provided in the Supplemental data, available online on the journal website.

### 2.2. CA inhibition assay

An SX.18V-R Applied Photophysics (Oxford, UK) stopped flow instrument has been used to assay the catalytic/inhibition of various CA isozymes^18^. Phenol Red (at a concentration of 0.2 mM) has been used as an indicator, working at an absorbance maximum of 557 nm, with 10 mM Hepes (pH 7.4) as a buffer, 0.1 M Na_2_SO_4_ or NaClO_4_ (for maintaining constant the ionic strength; these anions are not inhibitory in the used concentration), following the CA-catalysed CO_2_ hydration reaction for a period of 5–10 s. Saturated CO_2_ solutions in water at 25 °C were used as substrate. Stock solutions of inhibitors were prepared at a concentration of 10 mM (in DMSO–water 1:1, v/v) and dilutions up to 0.01 nM done with the assay buffer mentioned above. At least seven different inhibitor concentrations have been used for measuring the inhibition constant. Inhibitor and enzyme solutions were pre-incubated together for 10 min at room temperature prior to assay, in order to allow for the formation of the E–I complex. Triplicate experiments were done for each inhibitor concentration, and the values reported throughout the paper is the mean of such results. The inhibition constants were obtained by non-linear least squares methods using the Cheng–Prusoff equation, as reported earlier, and represent the mean from at least three different determinations. All CA isozymes used here were recombinant proteins obtained as reported earlier by our group^19^^,^^20^.

## 3. Results and discussion

### 3.1. Chemistry

**Scheme 1.**
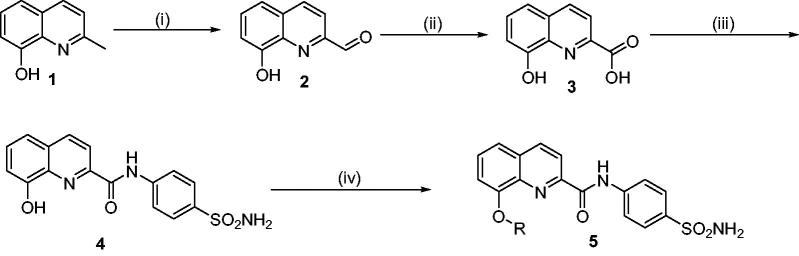
General synthetic route for the synthesis of 8-substituted quinoline-linked sulfonamide derivatives (**5a**–**h**). Reagents and conditions: (i) SeO_2_, 1,4-dioxane, 110 °C, 12 h, (ii) H_2_O_2_, Formic acid, 0 °C, 12 h, (iii) Sulfanilamide, HATU, DIPEA, DMF, 0 °C-rt, 12–15 h, and (iv) R-X, K_2_CO_3_, Acetone, r.t., 12–15 h.

A series of structurally diverse 8-substituted quinoline-2-carboxamides were synthesised according to general synthetic route as illustrated in Scheme 1. In brief, 8-hydroxy quinaldine (**1**) was treated with selenium dioxide to yield 8-hydroxyquinoline-2-carbaldehyde (**2**) which was further treated with H_2_O_2_ in the presence of formic acid to yield 8-hydroxyquinoline-2-carboxylic acid^21^ (**3**). This intermediate was further subjected to acid-amine coupling with sulfanilamide to give the amide product (**4**). This was further subjected to O-alkylation using various aliphatic and benzylic halides to afford the final products (**5a**–**h**). All the final products were confirmed using various analytical and spectral techniques.

### 3.2. CA inhibition studies

The newly synthesised 8-substituted quinoline-linked sulfonamide derivatives were evaluated for their inhibitory activity against the cytosolic isoforms hCA I and II, the membrane-bound isoform hCA IV and the transmembrane isoform hCA IX. Acetazolamide was used as a standard drug. The following structure–activity relationship (SAR) may be inferred from the inhibition data shown in Table 1:

**Table 1.** CA inhibition data with the synthesised compounds **5a**–**5h** and acetazolamide as standard drug, by a stopped flow CO_2_ hydrase assay.

*K*_I_ (nM)^a^					
Compound	R	hCA I	hCA II	hCA IV	hCA IX
**5a**	4-Nitrobenzyl	640.7	88.4	691.5	>10,000
**5b**	2-Bromobenzyl	444.4	85.7	3927	>10,000
**5c**	Benzyl	5290	8342	6410	>10,000
**5d**	3,5-Dimethylbenzyl	8126	8759	6757	>10,000
**5e**	3-Chlorobenzyl	6470	5175	806.7	>10,000
**5f**	2,5-Difluorobenzyl	6561	956.7	786.9	>10,000
**5g**	Prop-2-yn-1-yl	6914	5111	657.2	>10,000
**5h**	Methyl	61.9	33.0	1749	>10,000
**AAZ**	–	250	12.1	74	25.8

a Mean from three different assays, by a stopped flow technique (errors were in the range of ±5–10% of the reported values).

- The ubiquitous cytosolic isoform, hCA I, which is localised in the erythrocytes, gastrointestinal tract and eye, was inhibited from low to high nanomolar range, with the inhibition constants ranging from 61.9 to 8126 nM. The best inhibition constant was shown by compound **5h**, possessing a methyl substitution on the 8-OH group. It elicited an inhibition of more than four times compared to the standard **AAZ**, with a *K*_I_ of 61.9 nM.
- The cytosolic isoform, hCA II, which is also one of the physiologically relevant isoforms, was inhibited in a diverse manner by the compounds **5a**–**h**, with the inhibition constants ranging from 33.0 to 8759 nM. The most potent inhibitor was again found to be compound **5h**, with an inhibition constant of 33.0 nM. Compounds **5a** and **5b**, possessing a 4-nitro benzyl and 2-bromo benzyl substitution at 8-OH position, also showed low nanomolar inhibitory potencies with *K*_I_ values of 88.4 and 85.7 nM, respectively.
- The membrane-bound isoform, hCA IV, was inhibited with a moderate to weak inhibition profile by the compounds **5a**–**h**. The inhibition constants ranged from 657.2 to 6757 nM, as compared to the standard, **AAZ**, which showed a much stronger inhibition at 74 nM.
- The tumour-associated isoform, hCA IX, was not at all inhibited by the compounds **5a–h** (*K*_I_ > 10,000 nM).

## 4. Conclusions

A series of novel 8-substituted quinoline-linked sulfonamide derivatives **(5a–h)** were synthesised and assayed for inhibitory activity against a series of CA isoforms, namely, hCA I and II which are cytosolic, hCA IV which is membrane-bound and hCA IX which is tumour-associated isoform. Except for hCA IX, the synthesised compounds exhibited a variable degree of inhibition profiles for the other isoforms, ranging from low nanomolar to high nanomolar. Among all the compounds, compound **5h** exhibited low nanomolar inhibitory profiles for hCA I, with *K*_I_ 61.9 nM (wherein it proved to be four times more potent than the standard **AAZ**) and hCA II, with *K*_I_ of 33.0 nM. Compounds **5a** and **5b** showed inhibition constants of 88.4 and 85.7 nM respectively for hCA II. Apart from that, all the compounds showed moderate to high nanomolar inhibition for hCA I, II, and IV. Hence, compound **5h** can be developed further as a lead compound to design more effective hCA I and hCA II inhibitors.

## Supplementary Material

Supplemental Material
