# Supplementary material for: Synthesis and biological evaluation of novel 8-substituted quinoline-2-carboxamides as carbonic anhydrase inhibitors
Source: J Enzyme Inhib Med Chem. 2019 Jun 20;34(1):1172–7. doi: 10.1080/14756366.2019.1626376 (PMC6586119; doi:10.1080/14756366.2019.1626376)

SUPPORTING INFORMATION

Synthesis and biological evaluation of novel 8-substituted quinoline-2-carboxamides as carbonic anhydrase inhibitors

Pavitra S. Thacker[a], Pirpasha Shaikh[a], Andrea Angeli[b], Mohammed Arifuddin[a]* and Claudiu T. Supuran[b]*

[a]Department of Medicinal Chemistry, National Institute of Pharmaceutical Education and Research (NIPER), Balanagar, Hyderabad 500037, India

[b]Università degli Studi di Firenze, Neurofarba Dept., Sezione di Scienze Farmaceutiche e Nutraceutiche, Via Ugo Schiff 6, 50019 Sesto Fiorentino, Florence, Italy

^{1}H NMR Spectra of compound 6A; DMSO-d_6; 500 MHz

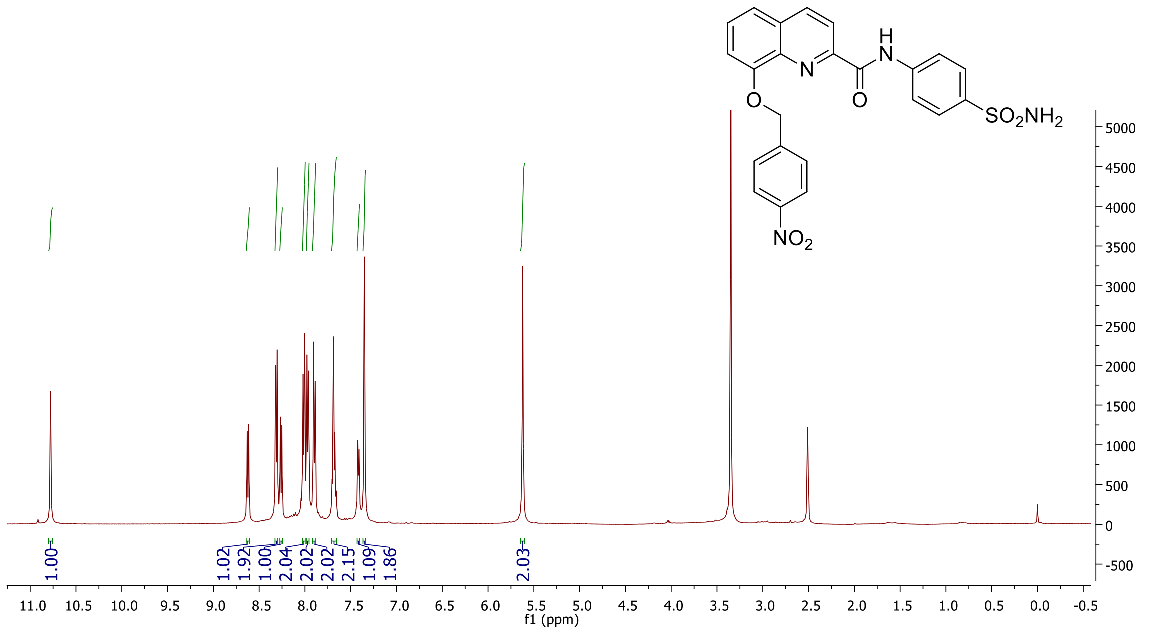

^{13}C NMR Spectra of compound 6A; DMSO-d_6; 125 MHz

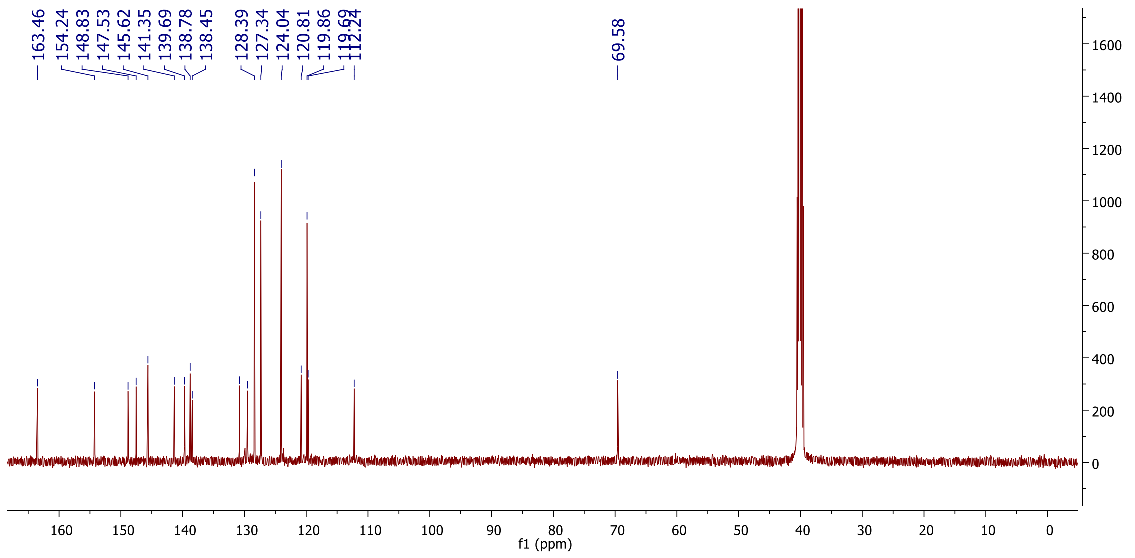

^{1}H NMR Spectra of compound 6B; DMSO-d_6; 500 MHz

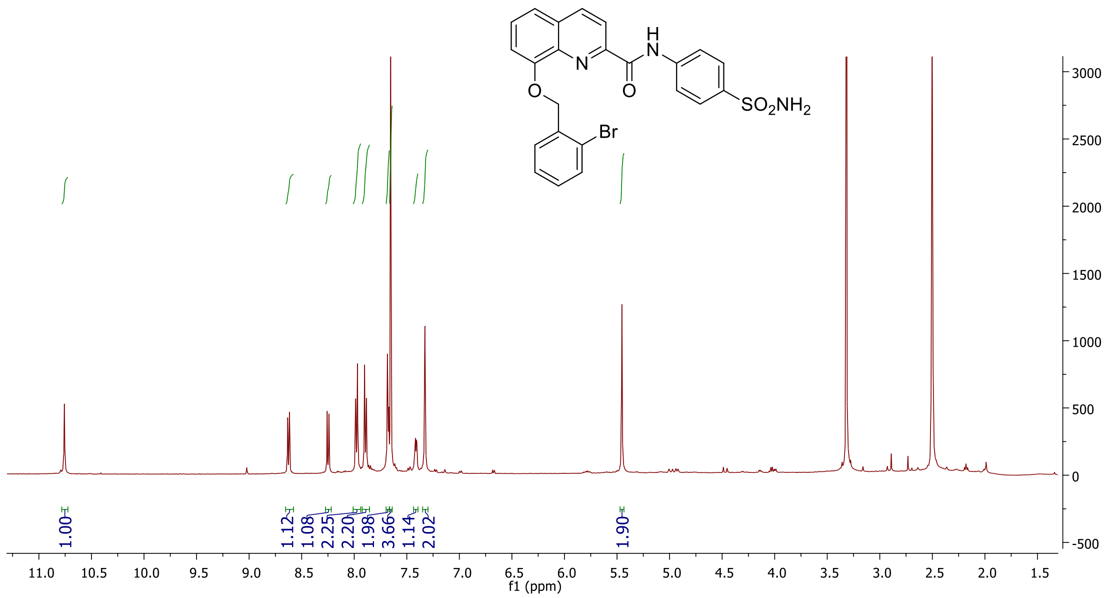

^{13}C NMR Spectra of compound 6B; DMSO-d_6; 125 MHz

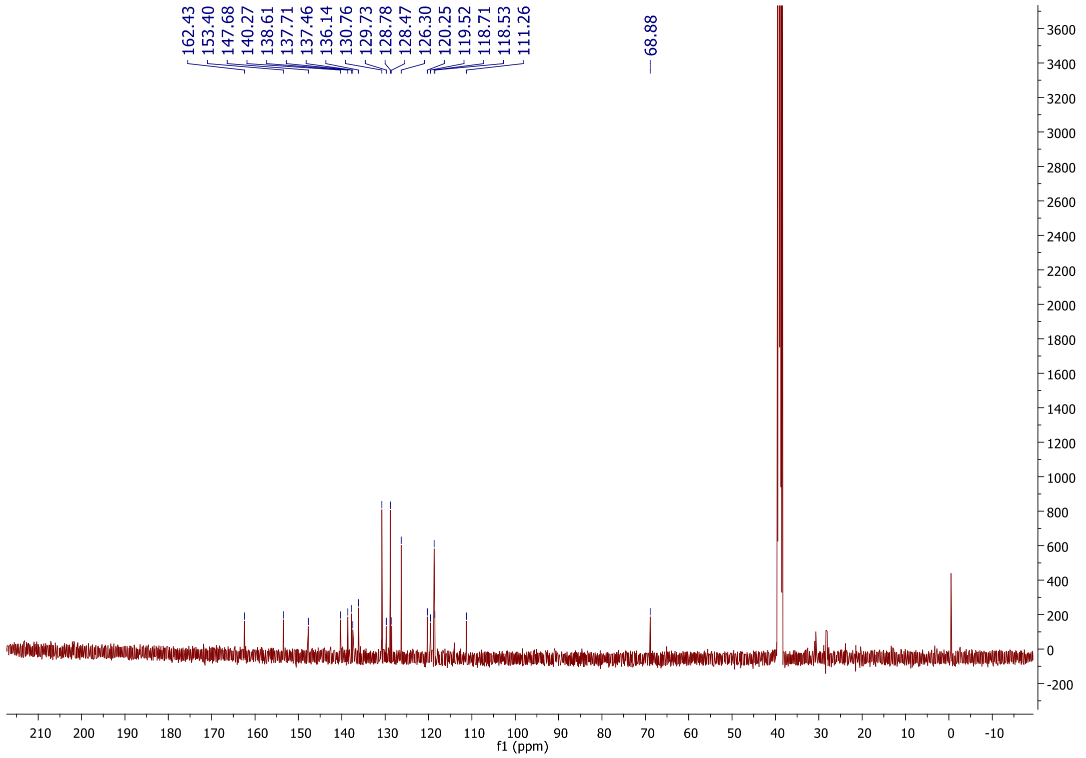

^{1}H NMR Spectra of compound 6c; DMSO-*d*$_6$; 500 MHz

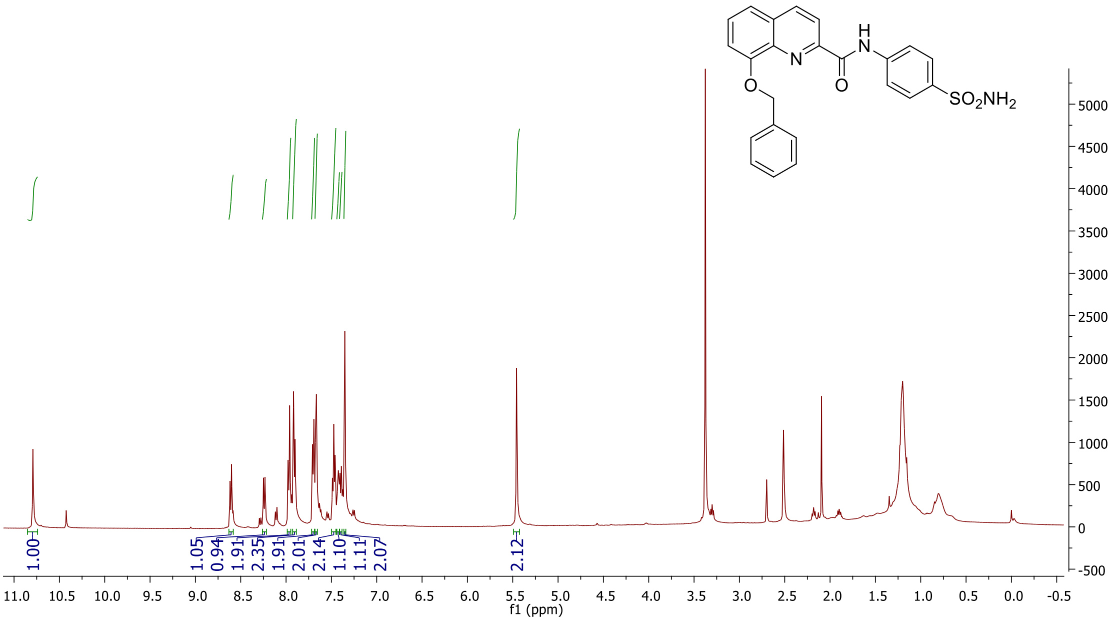

^{13}C NMR Spectra of compound 6c; DMSO-*d*$_6$; 125 MHz

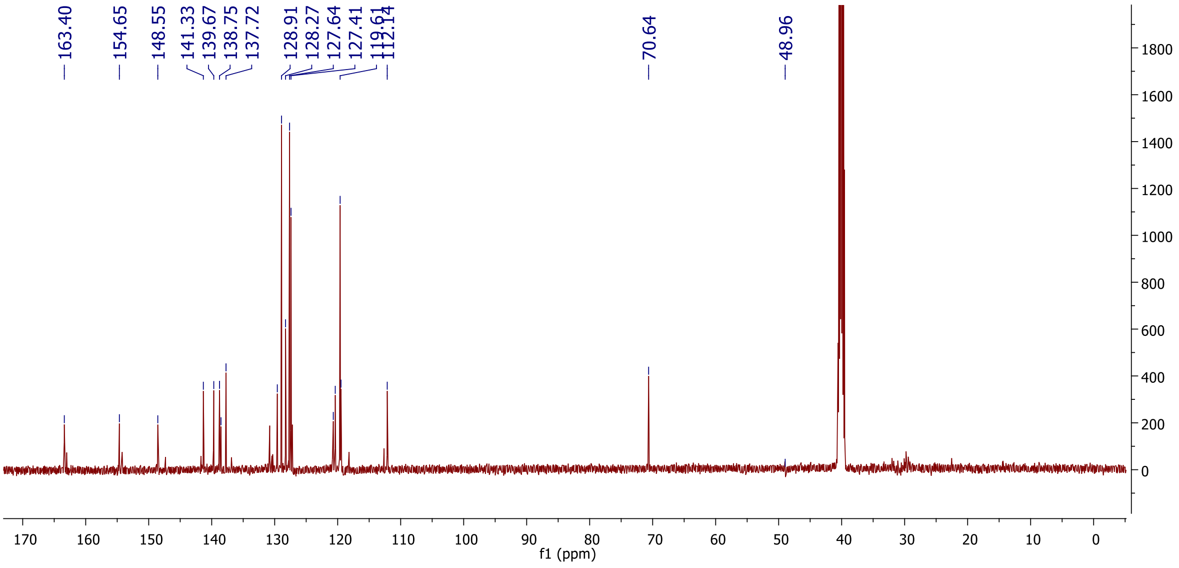

^{1}H NMR Spectra of compound 6D; DMSO-*d*$_6$; 500 MHz

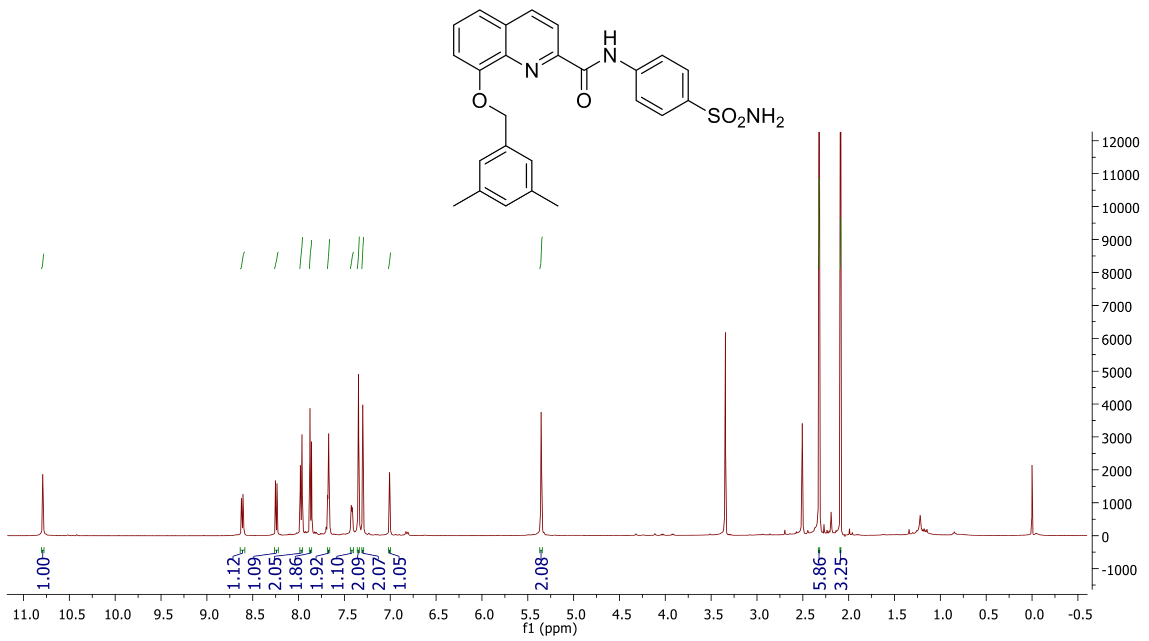

^{13}C NMR Spectra of compound 6D; DMSO-*d*$_6$; 125 MHz

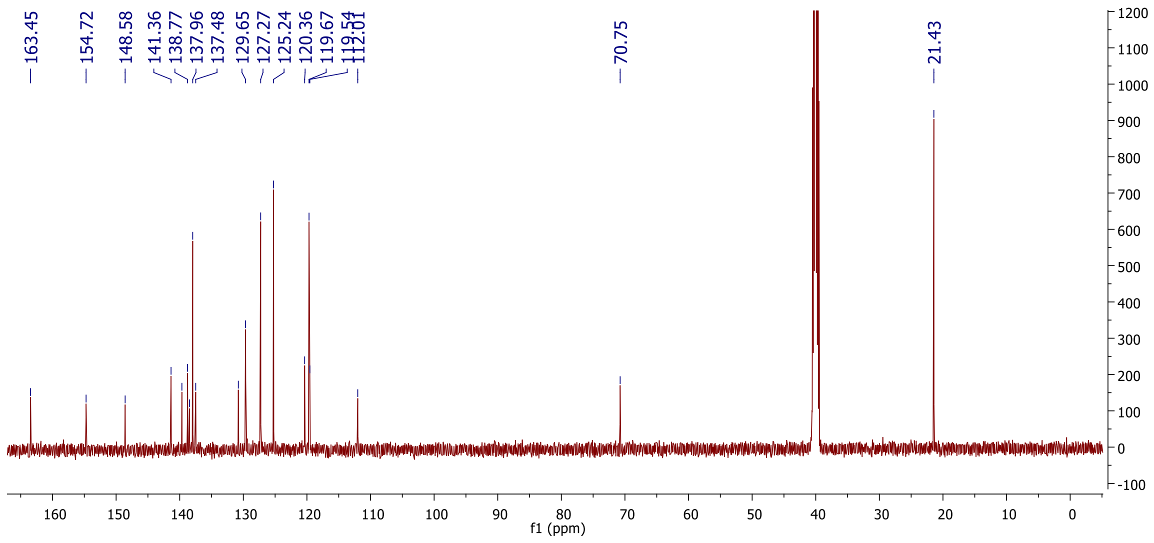

^{1}H NMR Spectra of compound 6E; DMSO-d_6; 500 MHz

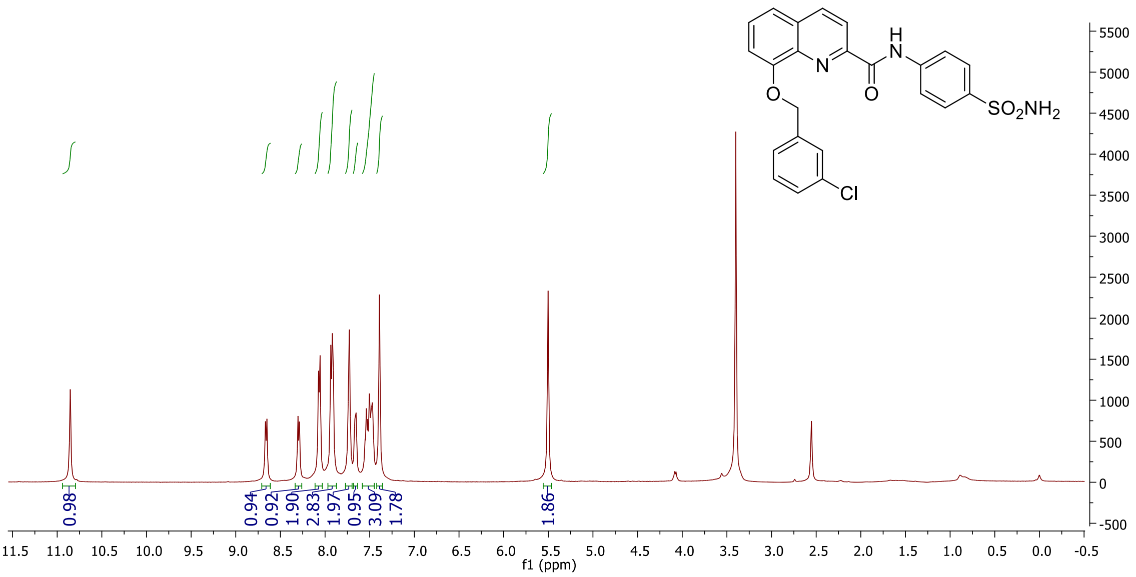

^{13}C NMR Spectra of compound 6E; DMSO-d_6; 125 MHz

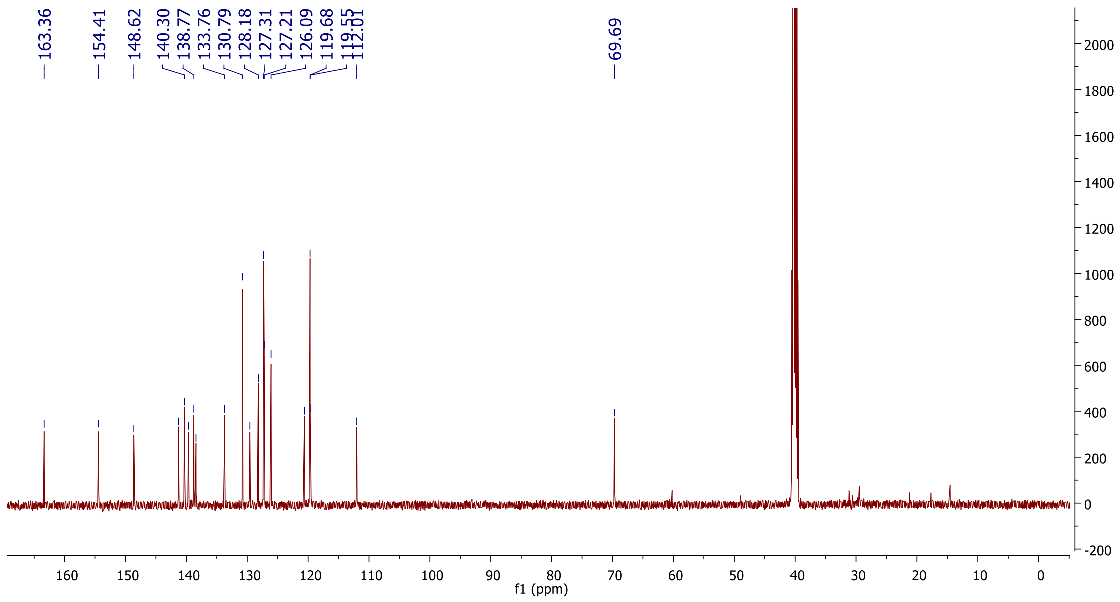

^{1}H NMR Spectra of compound 6F; DMSO-d_6; 500 MHz

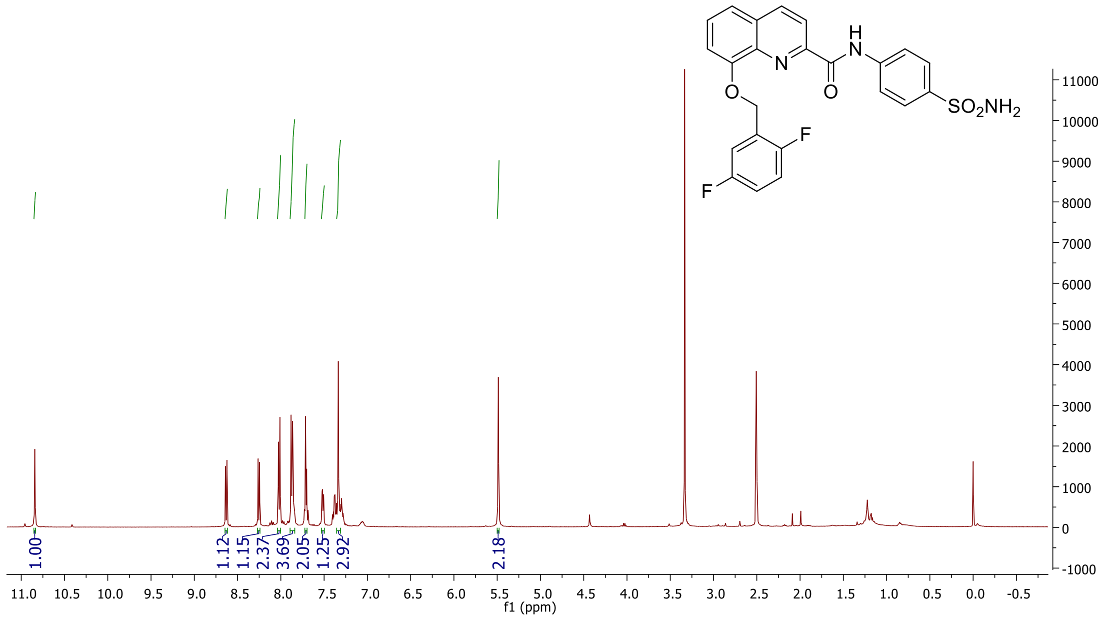

^{13}C NMR Spectra of compound 6F; DMSO-d_6; 125 MHz

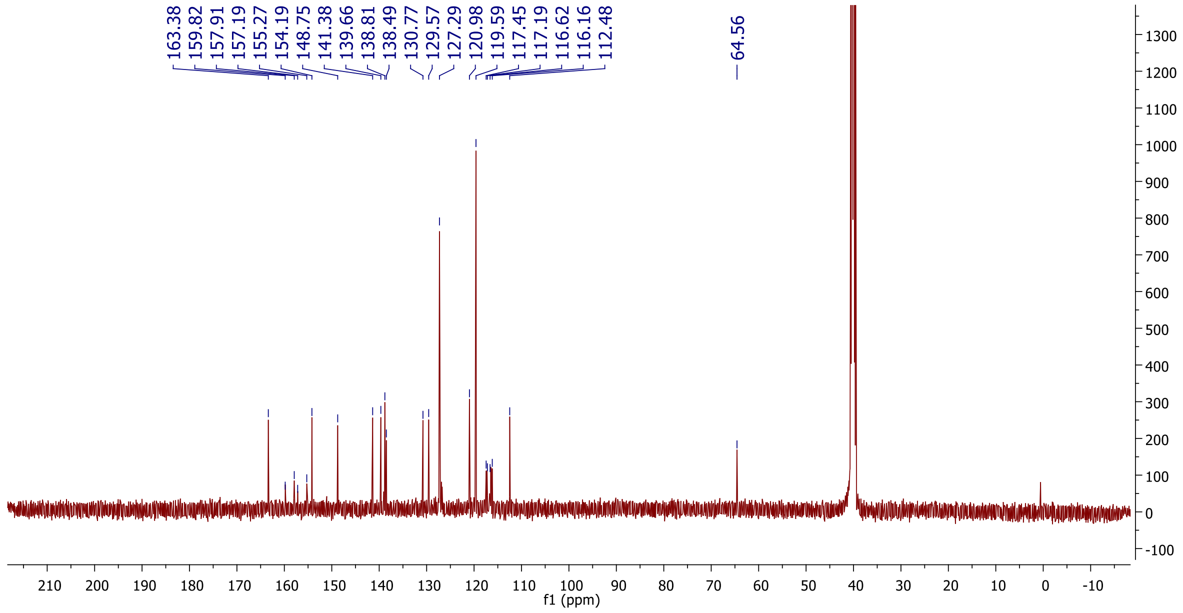

^{1}H NMR Spectra of compound 6g; DMSO-d_6; 500 MHz

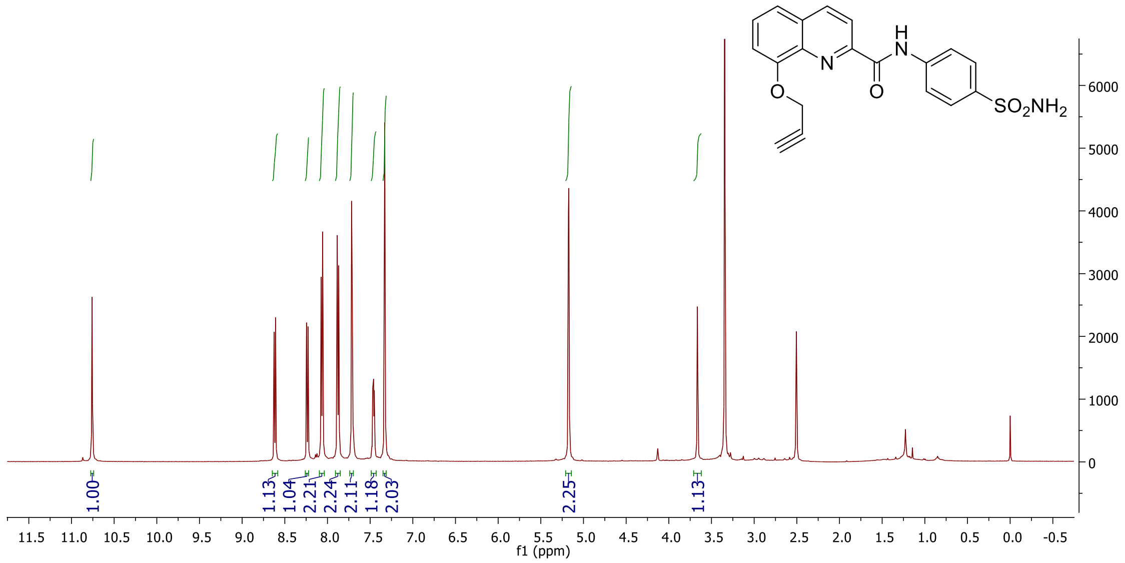

^{13}C NMR Spectra of compound 6g; DMSO-d_6; 125 MHz

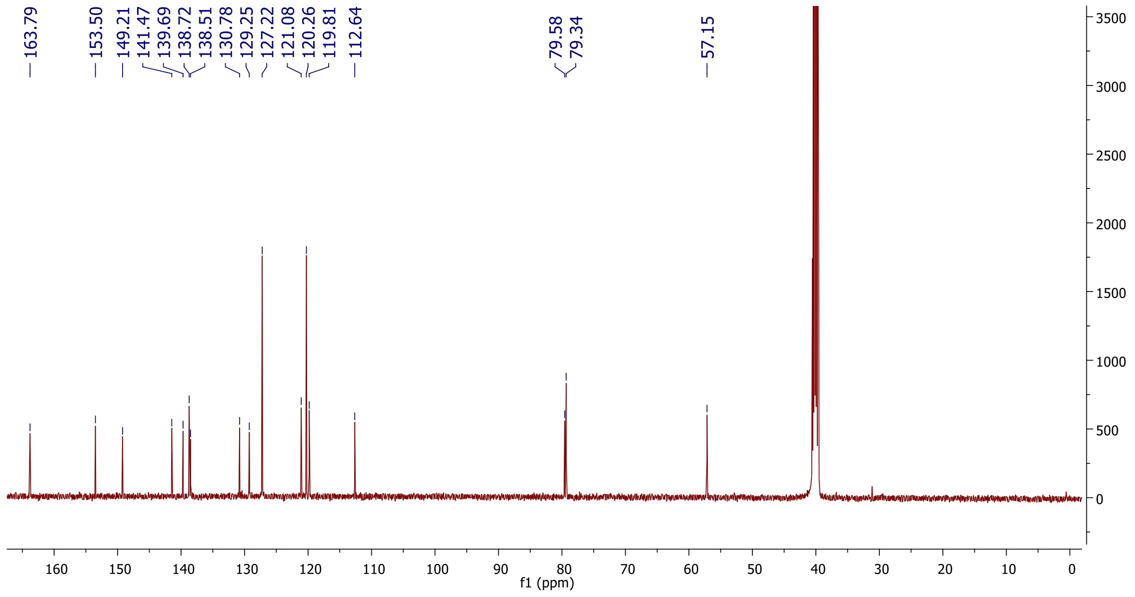

^{1}H NMR Spectra of compound 6h; DMSO-d_6; 500 MHz

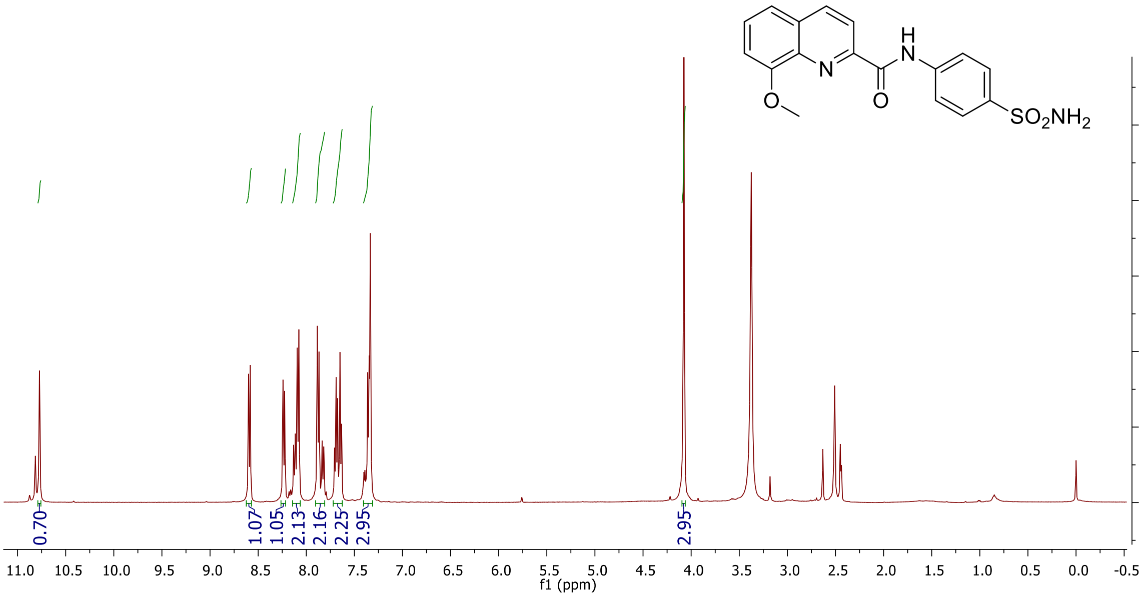

^{13}C NMR Spectra of compound 6h; DMSO-d_6; 125 MHz

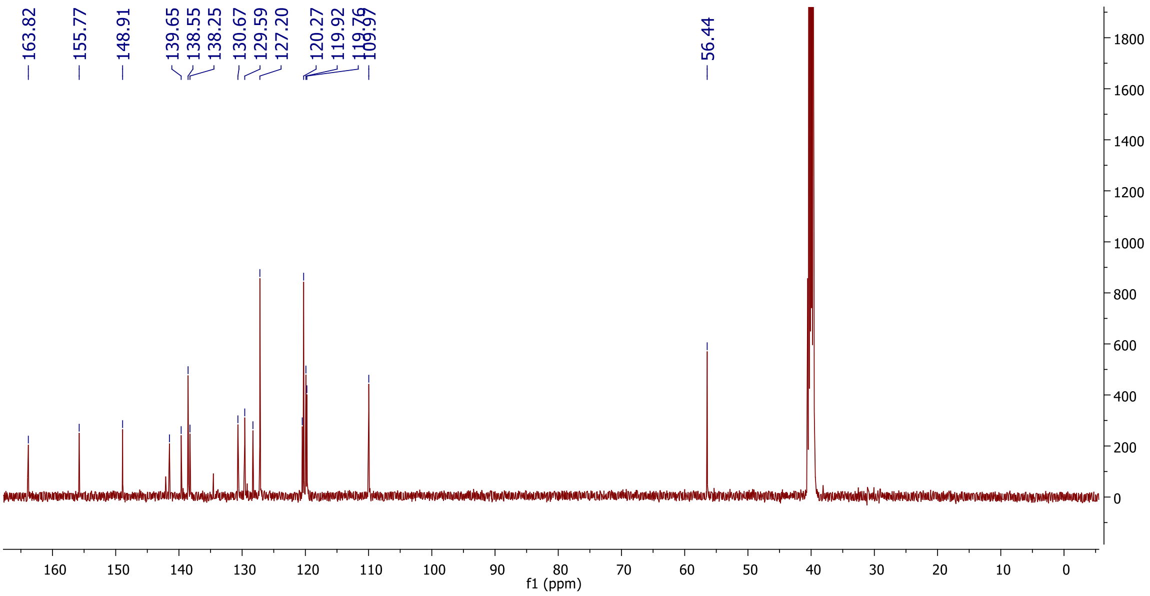

Supplement: Supplemental Material [file IENZ_A_1626376_SM4860.pdf]
